# Myeloid-Derived Suppressor Cells Modulate Immune Responses Independently of NADPH Oxidase in the Ovarian Tumor Microenvironment in Mice

**DOI:** 10.1371/journal.pone.0069631

**Published:** 2013-07-26

**Authors:** Heidi E. Godoy, A. Nazmul H. Khan, R. Robert Vethanayagam, Melissa J. Grimm, Kelly L. Singel, Nonna Kolomeyevskaya, Kevin J. Sexton, Anupama Parameswaran, Scott I. Abrams, Kunle Odunsi, Brahm H. Segal

**Affiliations:** 1 Department of Gynecology, Roswell Park Cancer Institute, Buffalo, New York, United States of America; 2 Department of Medicine, Roswell Park Cancer Institute, Buffalo, New York, United States of America; 3 Department of Immunology, Roswell Park Cancer Institute, Buffalo, New York, United States of America; 4 Department of Medicine, School of Medicine and Biomedical Sciences, University at Buffalo, Buffalo, New York, United States of America; Philipps University, Germany

## Abstract

The phagocyte NADPH oxidase generates superoxide anion and downstream reactive oxidant intermediates in response to infectious threat, and is a critical mediator of antimicrobial host defense and inflammatory responses. Myeloid-derived suppressor cells (MDSCs) are a heterogeneous population of immature myeloid cells that are recruited by cancer cells, accumulate locally and systemically in advanced cancer, and can abrogate anti-tumor immunity. Prior studies have implicated the phagocyte NADPH oxidase as being an important component promoting MDSC accumulation and immunosuppression in cancer. We therefore used engineered NADPH oxidase-deficient (p47*^phox−/−^*) mice to delineate the role of this enzyme complex in MDSC accumulation and function in a syngeneic mouse model of epithelial ovarian cancer. We found that the presence of NADPH oxidase did not affect tumor progression. The accumulation of MDSCs locally and systemically was similar in tumor-bearing wild-type (WT) and p47*^phox−/−^* mice. Although MDSCs from tumor-bearing WT mice had functional NADPH oxidase, the suppressive effect of MDSCs on *ex vivo* stimulated T cell proliferation was NADPH oxidase-independent. In contrast to other tumor-bearing mouse models, our results show that MDSC accumulation and immunosuppression in syngeneic epithelial ovarian cancer is NADPH oxidase-independent. We speculate that factors inherent to the tumor, tumor microenvironment, or both determine the specific requirement for NADPH oxidase in MDSC accumulation and function.

## Introduction

Inflammatory cells that constitute the cancer microenvironment can limit or stimulate tumor growth. In cancers that are responsive to immune targeting, cytotoxic T lymphocytes are the major effector cells mediating antigen-driven anti-tumor immunity. However, factors produced by the tumor and its microenvironment can abrogate anti-tumor immunity and facilitate local spread and metastasis. The balance between immune responses that inhibit versus facilitate tumor growth can predict clinical outcome. Therapeutic targeting of immune pathways that facilitate tumor escape may extend periods of disease-free progression and, potentially, overcome barriers to durable anti-tumor immunity.

Myeloid-derived suppressor cells (MDSCs) are a heterogeneous population of immature myeloid cells that are recruited by cancer cells and can accumulate both locally and systemically in advanced cancer. Mouse MDSCs are a heterogeneous myeloid population consisting of CD11b^+^Gr1^+^ cells. The two major MDSC sub-populations, granulocytic and monocytic, are defined based on the expression of Ly6G and Ly6C, the components of Gr1, and by their immunosuppressive activity. Granulocytic MDSCs are CD11b^+^Ly6G^+^Ly6C^low/neg^ and monocytic MDSCs are CD11b^+^Ly6G^neg^Ly6C^high^ [1], [2]. MDSCs can suppress anti-tumor responses through several mechanisms: suppression of CD4^+^ and CD8^+^ T cells by arginine and cysteine depletion, inhibition of T cell recruitment to tumor sites, inhibition of T cell-peptide-MHC interactions, skewing of the cytokine milieu toward type 2 or regulatory responses, and modulating NK and NKT responses [3]–[13]. In addition to their immunosuppressive properties, MDSCs can secrete factors (e.g., vascular endothelial growth factor (VEGF)) that enhance tumor growth, invasion, and metastasis [5], [14].

Epithelial ovarian cancer (EOC) is typically diagnosed at advanced stages. Even with optimal surgical debulking and chemotherapy, the vast majority of patients with advanced EOC will have progression of disease. However, there is considerable variability in progression-free survival and overall survival among patients with advanced EOC [15]. The role of immune surveillance in EOC was demonstrated by correlation of survival with tumor-infiltrating lymphocytes (TILs) [16]. Intraepithelial CD8^+^ TILs and a high CD8^+^/Treg ratio were associated with favorable prognosis in patients with EOC [17], [18]. Changes in the phenotype of tumor-infiltrating DCs influence EOC progression in mice [19]. MDSCs accumulate in the ascites of patients with advanced EOC and suppress T cell proliferation *ex vivo* [20]. These findings raise the potential for MDSCs in the EOC microenvironment as potential therapeutic targets. It is therefore important to understand mechanisms that lead to expansion and functional properties of MDSCs.

There is growing evidence that reactive oxidant intermediates (ROIs) and reactive nitrogen intermediates modulate the development of MDSCs. NADPH oxidase (NOX2) is the major source of ROIs in activated phagocytes. NADPH oxidase activation requires translocation of the cytoplasmic subunits p47*^phox^*, p67*^phox^*, and p40*^phox^* and rac to the membrane-bound flavocytochrome consisting of gp91*^phox^* and p22*^phox^* (phox, phagocyte oxidase). Following activation, NADPH oxidase converts molecular oxygen to superoxide anion that can be subsequently converted to downstream ROI metabolites (e.g., hydrogen peroxide). NADPH oxidase is critical for antimicrobial host defense and also modulates inflammatory responses [21]. Prior studies in mice have shown that NOX2 enhances MDSC differentiation and function [22], [23]. In the absence of NADPH oxidase, MDSCs lacked the ability to suppress T cell responses, and differentiated into mature macrophages and DCs [23]. In addition, *ex vivo* generation of human MDSCs from normal donor PBMCs by exposure to cytokines and tumor cell lines was associated with increased expression of NADPH oxidase constituent proteins [24], [25].Taken together, these observations point to NADPH oxidase potentially favoring tumor progression by augmenting MDSC accumulation and immunosuppression in the tumor microenvironment.

EOC is characterized by peritoneal implants, ascites, and accumulation of tumor-associated macrophages and MDSCs [26], [27]. The effect of NADPH oxidase in these myeloid cells on tumor progression and local immune responses is unclear. Our major goals were to delineate the role of NADPH oxidase in ovarian tumor progression and in modulating MDSC accumulation and function in murine EOC. We found that tumor progression was similar between WT and engineered NADPH oxidase-deficient mice. Granulocytic and monocytic MDSC accumulation in the peritoneum and spleen was similar between genotypes. Although MDSCs from tumor-bearing WT mice had functional NADPH oxidase, the suppressive effect of MDSCs on *ex vivo* stimulated T cell proliferation was NADPH oxidase-independent. We therefore conclude that in murine EOC, NADPH oxidase is dispensable for MDSC accumulation and immunosuppressive function. Understanding how the oxidative milieu modulates MDSC function, including NADPH oxidase-dependent and -independent pathways, may lead to novel ways to target these cells to enhance durable anti-tumor immunity.

## Materials and Methods

### Ethics statement

All mice were maintained under specific pathogen free conditions at the animal care facility at Roswell Park Cancer Institute and used in compliance with all relevant laws and institutional guidelines under a protocol approved by the Roswell Park Cancer Institute Institutional Animal Care and Use Committee.

### Mice

Mice with a targeted disruption of the p47*^phox^* gene (p47*^phox−/−^*) have a defective phagocyte NADPH oxidase (NOX2), rendering phagocytes incapable of generating measurable superoxide [28]. p47*^phox−/−^* mice are backcrossed 14 generations in the C57BL/6Ncr. A p47*^phox−/−^* mouse breeding colony is established at Roswell Park Cancer Institute (Buffalo, NY) and gp91*^phox−/−^* female mice were purchased from Jackson Labs (Bar Harbor, ME). Female mice (age 6–8 weeks) were used in all experiments. All mice were maintained under specific pathogen free conditions at the animal care facility at Roswell Park Cancer Institute and used in compliance with all relevant laws and institutional guidelines under a protocol approved by the Roswell Park Cancer Institute Institutional Animal Care and Use Committee.

### Mouse ovarian surface epithelial cancer (MOSEC) cells

The ID8 MOSEC line was derived from epithelial ovarian cells harvested from female C57BL/6 (H-2^b^) mice that were passaged *in vitro* [29]. Intraperitoneal injection of clonal lines established from late passage epithelial cells from syngeneic tumors in C57BL/6 mice results in ascites and peritoneal implants that mimic the human disease [29]. ID8 MOSEC cells (a kind gift from Dr. P. Terranova, University of Kansas Medical Center, Kansas City, USA) were cultured in RPMI 1640 media with heat-inactivated FBS (10%), L-glutamine (2 mM), HEPES (25 mM), Sodium Pyruvate (1 mM), 2-Mercaptoethanol (50 µM), Penicillin-Streptomycin (100 U/ml), and non-essential amino acids.

### Tumor administration

Mice were administered intraperitoneal ID8 MOSEC cells (5×10^6^ cells in 200 µL PBS/mouse). For survival experiments, mice were monitored up to 150 days, and euthanized based on abdominal distention, ruffled fur, lethargy or inability to ambulate. In separate experiments, mice were sacrificed on day 42 or 90 after tumor administration for assessment of immunological endpoints. Day 42 corresponds to low tumor burden that models minimal residual disease in patients, and day 90 corresponds to advanced tumor burden.

### Isolation and processing of mouse cells

Following sacrifice, peritoneal exudate cells (PECs) were collected by peritoneal lavage with PBS (5–8 ml, containing 1% FBS and 0.5 mM EDTA). PECs were subjected to red cell lysis with ACK buffer, followed by washing. Cell-free supernatants from peritoneal lavage were frozen for subsequent cytokine measurements. Lymph nodes and spleens were also harvested at sacrifice, and single cell suspensions were subjected to red cell lysis and washing. Isolated PECs, splenocytes and lymph node cells were either used within 24 h of harvest for flow cytometry and functional studies or frozen in liquid nitrogen in media containing 20% FBS and 5% DMSO.

### Flow cytometry

Flow cytometry analysis was conducted on a FACScan (Becton Dickinson, Franklin Lakes, NJ). R-Phycoerythrin conjugated anti-mouse Ly6G and CD8, FITC conjugated anti-mouse Ly6C (BD Biosciences, San Jose, CA), APC conjugated anti-mouse CD11b, Pacific Blue conjugated anti-mouse CD4, and eFlour 450 conjugated anti-mouse F4/80 (eBioscience, San Diego, CA) mAb, and respective isotype controls were used. Forward scatter versus side scatter gating was set to include all non-aggregated cells. Gating on CD11b^+^ cells, the proportion of granulocytic MDSCs (Ly6G^+^Ly6C^low^), monocytic MDSCs (Ly6C^+^Ly6G^−^), and macrophages (F4/80^+^) was determined [2].

### Functional assays

MDSC function was evaluated based on suppression of stimulated T cell proliferation in co-culture experiments. Myeloid cells from tumor-bearing mice were purified with anti-CD11b magnetic beads using autoMACS according to the manufacturer's protocol (Miltenyi Biotec Inc., Auburn, CA). Granulocytic MDSCs were column-purified using anti-Ly6G magnetic beads. Following column separation, the purity of cell fractions was analyzed microscopically by Diff-Quick-stained cytospins (Fisher Scientific, Kalamazoo, MI,) and by flow cytometry.

Splenocytes from non-tumor-bearing C57BL/6 female mice were used as naïve T cell targets. Following red cell lysis and washing, splenocytes were incubated with 5 µM carboxyfluoresceindiacetate succinimidyl ester (CFSE; Invitrogen, Grand Island, NY) in PBS for 8 min as previously described [30]. Cells (2.5 or 5×10^5^ cells/well) were cultured in triplicate in 96-well plates coated with anti-CD3 (10 µg/ml) mAb (BD Biosciences) and B7.1 antigen (0.5 µg/ml) (R&D Systems Inc., Minneapolis, MN). Equal numbers of magnetically separated CD11b^−^, CD11b^+^, or Ly6G^+^ cells isolated from tumor-bearing mice were added. After 72 h of co-culture, cells were collected, labeled with anti-CD4 and anti-CD8 mAb, and analyzed by flow cytometry. The proliferation of CFSE-labeled CD4^+^ and CD8^+^ T cells was evaluated by quantification of CFSE dilution staining as described [30]. The primary endpoint was the proportion of CFSE-loaded CD4^+^ and CD8^+^ T cells undergoing ≥1 replication.

### Cytokine analysis

Cell-free supernatants from peritoneal ascites were collected from ID8 MOSEC-bearing mice at day 90. Cytokine and VEGF concentrations were measured by ELISA according to the manufacturer's instructions (BD Biosciences).

### Reactive oxidant production

To measure the production of ROIs by granulocytic MDSCs, the oxidation sensitive dye H_2_DCFDA (Invitrogen) was used. PECs isolated from MOSEC-bearing WT and p47*^phox−/−^* mice at day 90, were stained with anti-CD11b and anti-Ly6G, and then incubated in 1% FBS-containing PBS at room temperature in dark with or without H_2_DCFDA (10 µM; Invitrogen) for 30 min. After washing, cells were incubated with or without PMA (50 nM) for 15 min, then transferred to ice, and immediately analyzed by flow cytometry.

### Statistical Analysis

Time to euthanasia was plotted using Kaplan-Meier curves and analyzed using the log-rank method. For immunologic endpoints, comparisons between two groups were assessed by student's t-test or Mann-Whitney if data were not normally distributed. Statistical analysis was performed using Graph Pad Prism 5 software.

## Results

### NADPH oxidase does not affect overall survival in mice with ovarian cancer

**Figure 1.Time:**
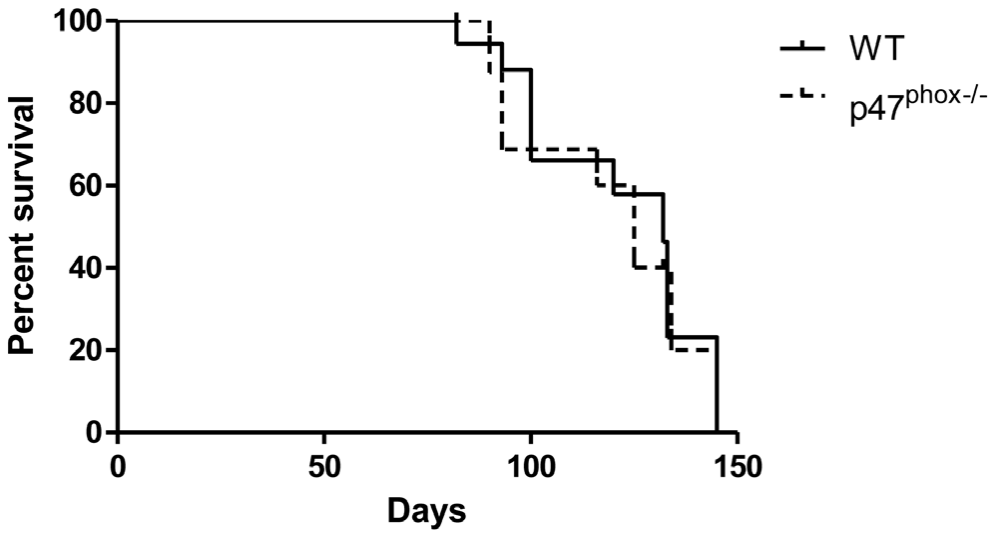
to tumor progression requiring euthanasia is not altered by NADPH oxidase. Kaplan-Meier plots of WT and NADPH oxidase-deficient (p47*^phox−/−^*) mice (10 mice/group) showed similar survival after i.p. MOSEC challenge (log-rank, p = 0.25).

To evaluate the role of NADPH oxidase in regulating ovarian tumor growth, we challenged WT and NADPH oxidase-deficient p47*^phox−/−^* mice with intraperitoneal MOSEC. Time to progression requiring euthanasia was similar in WT and p47*^phox−/−^* mice (Figure 1). Because phagocyte oxidase proteins could have NADPH oxidase-independent signaling, we evaluated survival following tumor challenge in another NADPH oxidase-deficient model (gp91*^phox−/−^*) [31], and found no significant difference compared to WT mice (data not shown).

To assess tumor progression prior to overt disease requiring euthanasia, WT and p47*^phox−/−^* mice were sacrificed either at day 42 (modeling minimal residual disease) or day 90 (modeling advanced disease) after MOSEC administration. At day 42, there was no detectable ascites, but there was variable appearance of tumor nodules measuring ∼1–5 mm in the peritoneal cavity of WT and p47*^phox−/−^* mice. At day 90, both genotypes had ascites with extensive tumor implants. There was no obvious effect of genotype at either time point based on visual inspection. These results show that NADPH oxidase does not have a major role in modulating the progression of ovarian tumor burden.

### Effect of NADPH oxidase in modulating MDSCs in the ovarian tumor microenvironment and systemically

**Figure 2:**
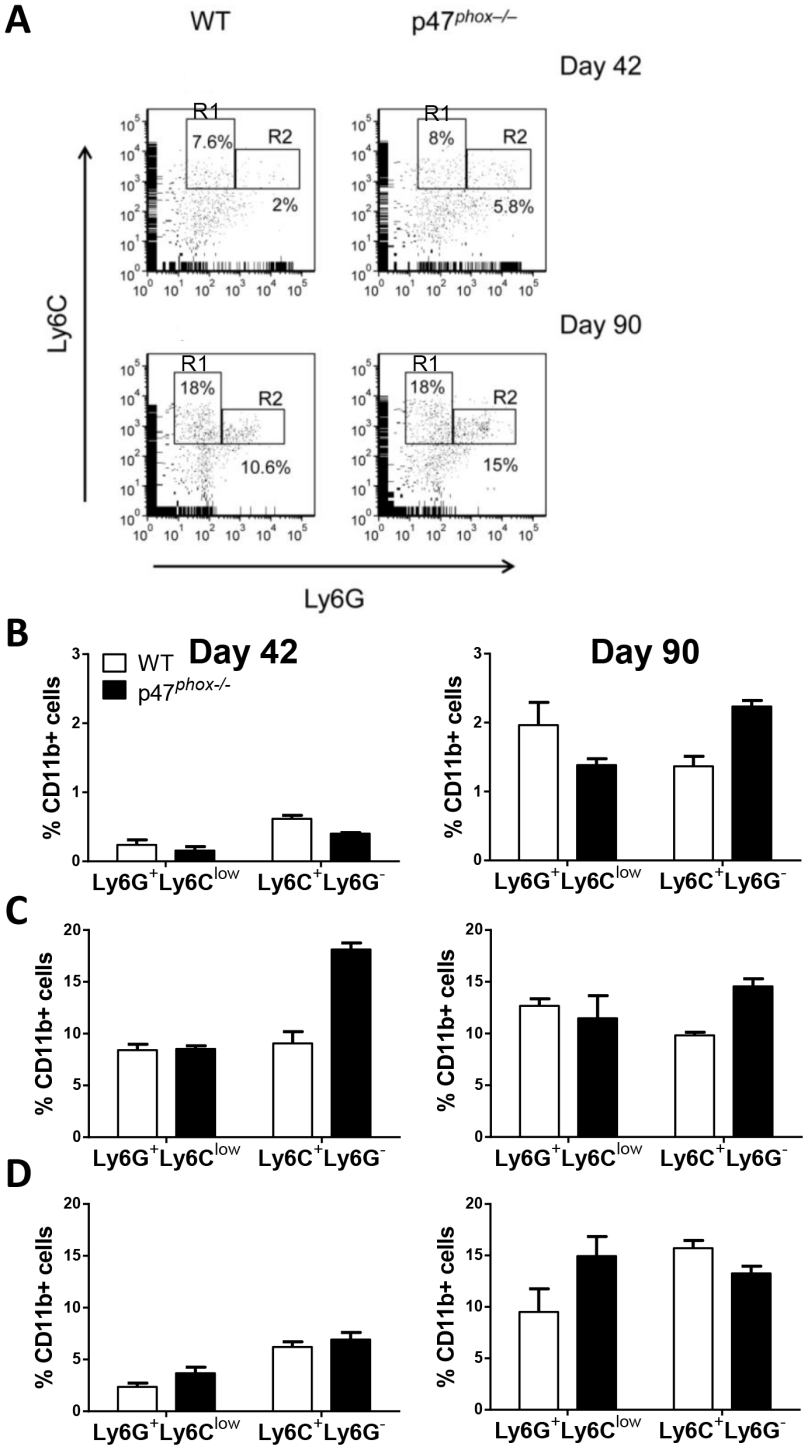
Effect of NADPH oxidase in local and systemic accumulation of MDSCs in tumor-bearing mice. A) Representative quantification of MDSCs. Splenocytes from WT and p47*^phox−/−^* mice at day 42 and 90 after MOSEC administration were analyzed for MDSC accumulation. Gating on myeloid (CD11b^+^) cells, the proportion of monocytic MDSCs (R1; Ly6C^+^Ly6G^−^) and granulocytic MDSCs (R2; Ly6G^+^Ly6C^Low^) significantly increased at day 90 versus day 42. All gates were set based on isotypes. This approach was used to quantify MDSCs in PECs, lymph nodes, and spleens. B) Proportion of MDSCs in myeloid PECs on day 42 and 90. The proportion with granulocytic and monocytic MDSC markers was greater in advanced (day 90) versus early (day 42) stage tumor burden in both genotypes. C) In draining lymph nodes, there was a trend toward increased monocytic MDSC accumulation in p47*^phox−/−^* versus WT mice at day 42 but not at day 90. There was no effect of NADPH oxidase on granulocytic MDSC accumulation at either time point. D) In spleens, there was an increased accumulation of MDSCs, particularly granulocytic MDSCs, in mice with advanced versus early disease, but no effect of mouse genotype. Data (± SEM) are from at least 3 mice per genotype per time point, and are representative of 3 separate experiments. Comparison between genotypes: p = NS.

NADPH oxidase could potentially have multiple effects on the tumor microenvironment that either promote or inhibit tumor progression, including modulation of the cytokine milieu, inflammatory cell recruitment, and antigen display and cross-presentation [32]–[37]. We undertook studies to evaluate the specific effects of NADPH oxidase on MDSC accumulation and function following MOSEC challenge. We evaluated MDSC accumulation in the peritoneal tumor microenvironment, draining lymph nodes (para-aortic and inguinal), and spleens at days 42 and 90 after MOSEC challenge in WT and NADPH oxidase-deficient mice. A well-defined granulocytic MDSC population (CD11b^+^Ly6G^+^Ly6C^low^) was observed in PECs and systemically in WT and p47*^phox−/−^* mice that increased in relation to tumor burden (Figure 2). We did not observe a well-defined CD11b^+^Ly6G^−^Ly6C^high^ population that characterized monocytic MDSCs in other tumor-bearing models [2]; therefore the monocytic MDSC population was defined based on CD11b^+^Ly6G^−^Ly6C^+^ expression (Figure 2A), realizing that this may contain a mixed population of monocytic cells.

In non-tumor-bearing mice, virtually all of the peritoneal myeloid cells were resident macrophages (CD11b^+^F4/80^+^) (data not shown). In MOSEC-bearing mice, tumor cells were the predominant cell population in peritoneal lavage fluid. Gating on peritoneal myeloid cells (CD11b^+^), the proportion with granulocytic and monocytic MDSCs was greater in advanced (day 90) versus early (day 42) stage tumor burden (Figure 2B). In particular, the proportion of peritoneal granulocytic MDSCs was 8 to 9-fold greater at day 90 versus day 42. In draining lymph nodes, there was no consistent effect of tumor burden on the proportion of MDSCs (Figure 2C). In spleens, there was an increased accumulation of MDSCs, particularly granulocytic MDSCs, in mice with advanced versus early disease (Figure 2D). To our surprise, NADPH oxidase deficiency had no significant impact on the accumulation of granulocytic or monocytic MDSCs at early or advanced disease. Together, these data show that NADPH oxidase does not regulate MDSC accumulation in the local tumor microenvironment or systemically in murine EOC.

**Figure 3:**
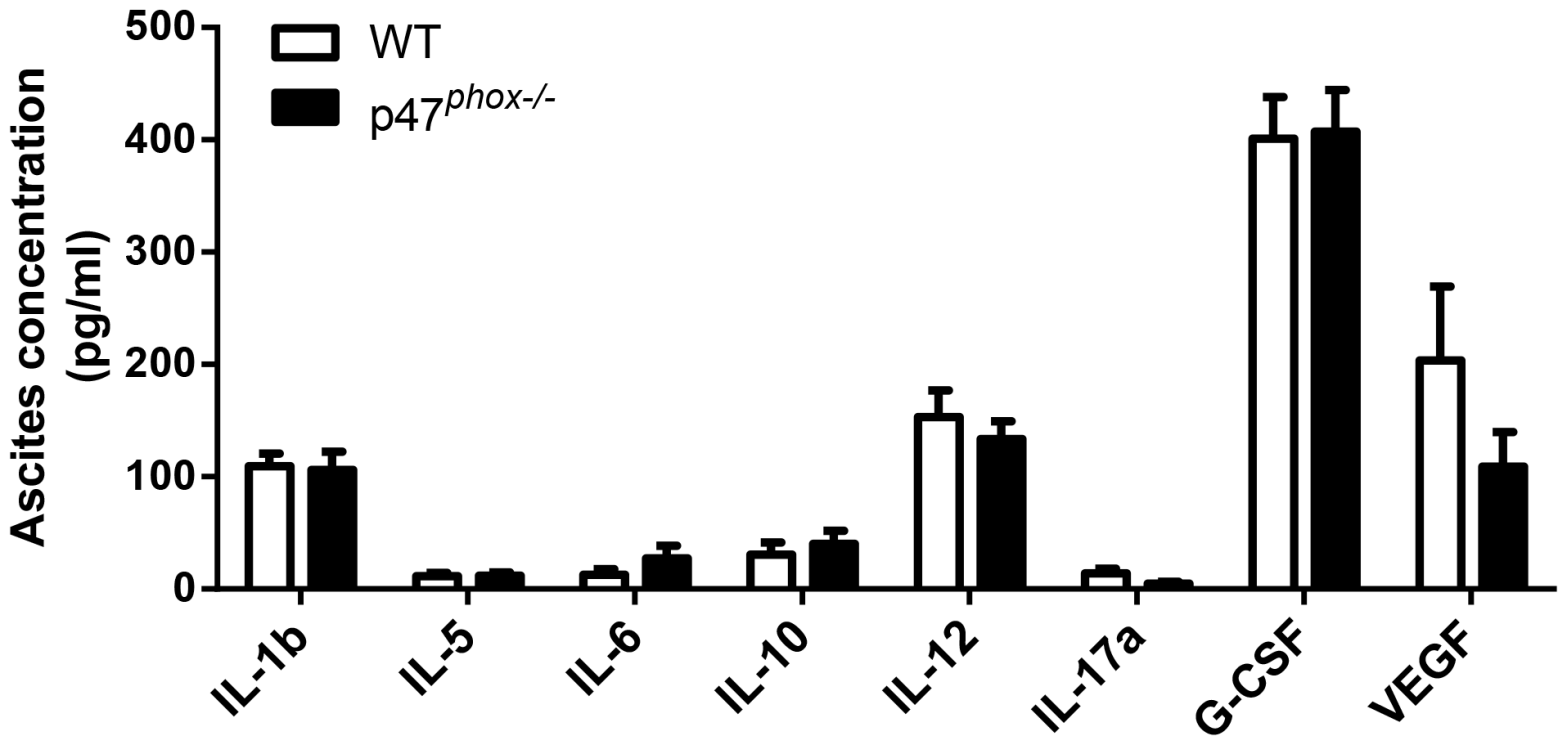
Role of NADPH oxidase in cytokine production in the ovarian tumor microenvironment. Cell-free supernatants collected from ascites from WT and p47*^phox−/−^* mice at day 90 after MOSEC administration were analyzed by ELISA for pro-inflammatory cytokines, G-CSF, and VEGF. N = 8 mice per genotype. Comparison between genotypes: p = NS.

Both the tumor and inflammatory cells in the tumor microenvironment can modulate cytokine responses mediating MDSC accumulation and function. Since NADPH oxidase can have a key role in modulating cytokine responses to microbes and microbial products [36] and in angiogenesis [38], we evaluated whether NADPH oxidase regulates inflammatory mediators in the tumor microenvironment. We found that cytokine and VEGF concentrations in ascites (day 90) were similar between WT and p47*^phox−/−^* mice (Figure 3). Thus, in the MOSEC tumor microenvironment, NADPH oxidase does not have a significant effect on modulation of mediators produced by MDSCs nor is it likely to influence MDSC recruitment. A limitation of this model is that we cannot distinguish tumor-derived from host-derived products in ascites.

### Peritoneal granulocytic MDSCs from WT MOSEC-bearing mice have intact p47^phox^-dependent NADPH oxidase function

**Figure 4:**
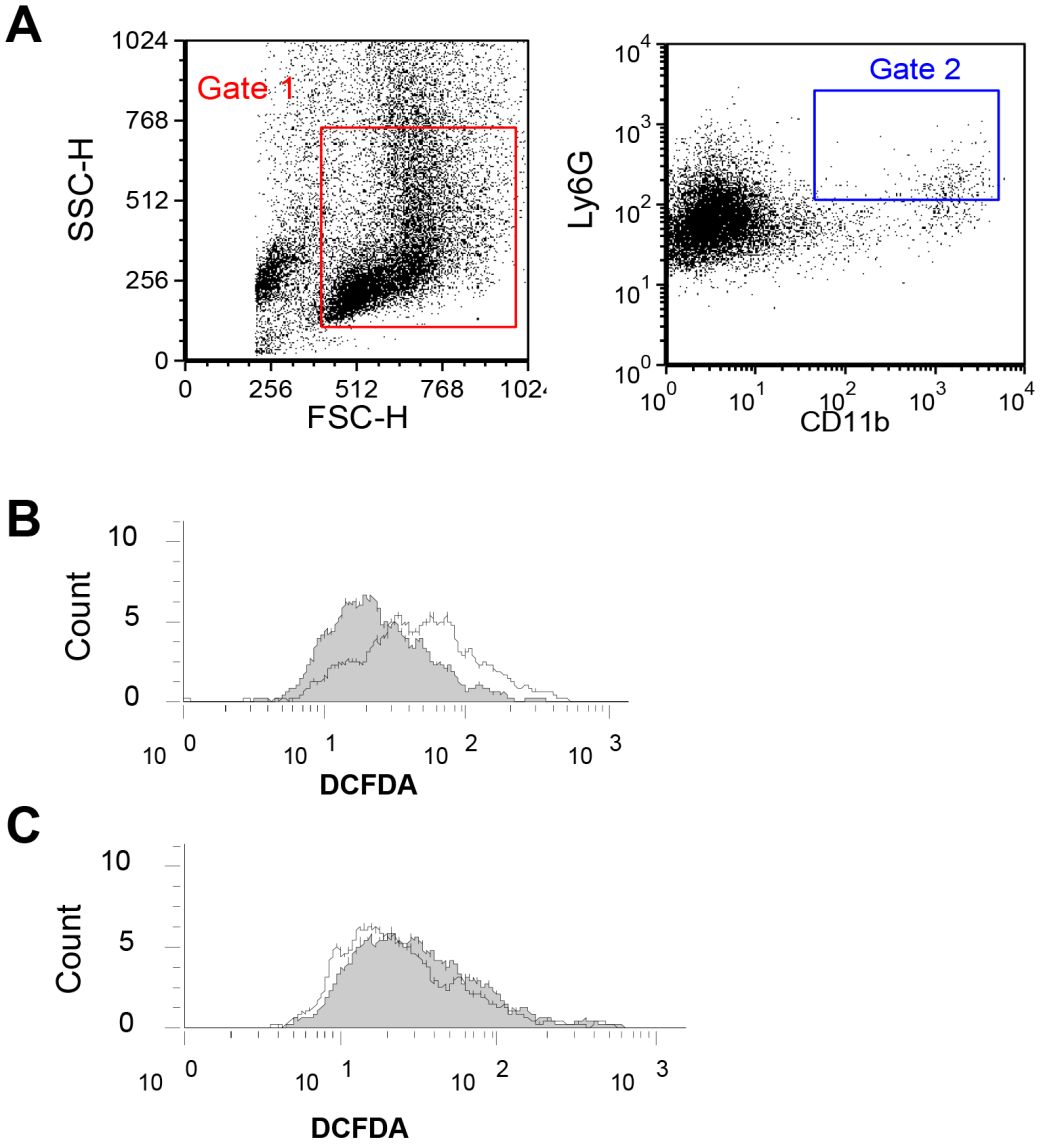
The p47*^phox^* component is required for NADPH oxidase activity in granulocytic MDSCs. PECs from WT and p47*^phox−/−^* mice harvested at day 90 after MOSEC challenge were stimulated with PMA, and intracellular ROI production in CD11b^+^Ly6G^+^ cells was assessed by H_2_DCFDA fluorescence. A) Gating on all non-aggregated cells (Gate 1), CD11b^+^Ly6G^+^ cells (Gate 2) were defined using respective isotype controls. B and C) Stimulated ROI production was detectable in WT (B), but not in NADPH oxidase-deficient (C), granulocytic MDSCs. White plot = PMA stimulation; shaded plot = no-stimulation. Results are representative of two experiments.

We next assessed whether NADPH oxidase activity is intact in MDSCs from tumor-bearing mice. Peritoneal exudate cells harvested at day 90 after MOSEC challenge from WT and p47*^phox−/−^* mice were stimulated with PMA (50 nM for 15 min), and intracellular ROI production in granulocytic MDSCs (CD11b^+^Ly6G^+^) was assessed by H_2_DCFDA fluorescence (Figure 4A). WT granulocytic MDSCs had intact NADPH oxidase activity (Figure 4B), whereas ROI generation was absent in p47*^phox−/−^* cells (Figure 4C). We did not detect ROI generation in cultured MOSEC cells stimulated with PMA for 15 or 60 min (not shown), indicating that these cells are unlikely to be a source of ROIs in tumor-bearing mice. Together, these results point to p47*^phox^* being required for NADPH oxidase activity in granulocytic MDSCs, but not significantly influencing MDSC accumulation in MOSEC-bearing mice.

### T cell suppression by ovarian tumor induced-MDSCs is independent of NADPH oxidase

**Figure 5:**
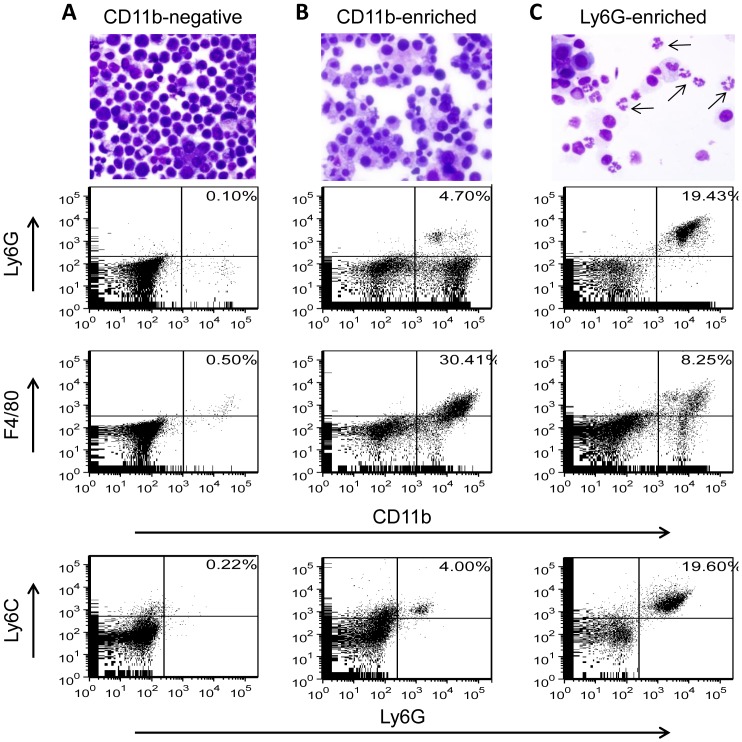
Analysis of peritoneal exudate cells from MOSEC-bearing mice after enrichment for myeloid cells and granulocytic cells. Myeloid cells and granulocytic cells from PECs of MOSEC-bearing WT and p47*^phox−/−^* mice were column-purified using anti-CD11b and anti-Ly6G, respectively. Analysis of post-enriched fractions showed concordance between cytology and flow cytometry. Representative analysis of PECs from p47^phox−/−^ mice collected on 90 after MOSEC administration is shown. A) The CD11b-negative fraction contained a preponderance of tumor cells (based on cytology), while myeloid cells were rare. B) The CD11b-enriched fraction contained a mixed myeloid cell population, with a preponderance of macrophages based on cytology and surface markers (CD11b^+^F4/80^+^). C) The Ly6G-enriched fraction contained a preponderance of granulocytic cells (arrows), with the majority of myeloid cells expressing granulocytic MDSC markers (CD11b^+^Ly6G^+^Ly6C^low^).

MDSCs are defined based on both surface marker expression and immunosuppressive function. Since NADPH oxidase did not significantly affect MDSC accumulation in MOSEC-bearing mice, we evaluated whether NADPH oxidase modulates MDSC function, focusing on granulocytic MDSCs. CD11b^+^ cells and Ly6G^+^ cells were isolated by magnetic separation from PECs of WT and p47*^phox−/−^* mice harvested at day 42 or 90 after MOSEC administration. Cytology and flow cytometry analysis of fractionated PECs showed concordant results, with similar findings between WT and p47*^phox−/−^* mice. Representative results from PECs harvested at day 90 are shown in Figure 5. The CD11b-negative fraction consisted predominantly of tumor cells, with sparse numbers of myeloid cells (<1%) (Figure 5A). The CD11b-enriched fraction was comprised principally of macrophages (CD11b^+^F4/80^+^), but also contained granulocytic and monocytic MDSCs (Figure 5B). The Ly6G-enriched fraction contained a mixed population of cells, with granulocytic MDSCs (CD11b^+^Ly6G^+^Ly6C^low^) being the predominant myeloid cell population. Cytology confirmed the presence of cells with a distinct granulocytic morphology in the Ly6G^+^ enriched fraction (Figure 5C).

**Figure 6:**
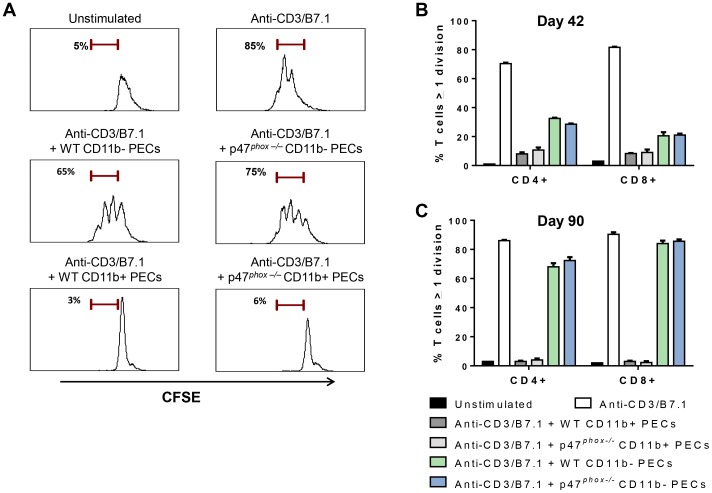
Myeloid peritoneal cells in MOSEC-bearing mice suppress T cell proliferation independently of NADPH oxidase. Purified myeloid (CD11b^+^) PECs from MOSEC-bearing WT and p47*^phox−/−^* mice were co-cultured with CFSE-labeled splenocytes from naïve mice (E∶T ratio: 1∶1) in anti-CD3/B7.1-coated plates. After 72 h of culture, CD4^+^ and CD8^+^ T cell proliferation was assessed based on CFSE dilution as described in methods. A) Representation histograms showing that CD11b^+^, but not CD11b^−^, PECs from MOSEC-bearing WT and p47*^phox−/−^* mice (day 90) completely suppress anti-CD3/B7.1-stimulated CD4^+^ T cells proliferation. Anti-CD3/B7.1 stimulated and unstimulated CD4^+^ T cells are used as positive and negative controls, respectively. B) CD11b-enriched PECs from MOSEC-bearing WT and p47*^phox−/−^* mice at day 42 equally suppress both CD4^+^ and CD8^+^ T cell proliferation. In contrast, CD11b^−^ PECs from the same mice incompletely suppressed anti-CD3/B7.1-stimulated T cell proliferation. C) CD11b-enriched PECs from MOSEC-bearing WT and p47*^phox−/−^* mice at day 90 completely suppressed T cell proliferation while the CD11b-negative fraction had no significant effect on T cell proliferation. Myeloid PECs collected on day 42 were pooled because of limited cell number, while non-pooled PECs from individual mice were analyzed from day 90 harvests. Individual experiments were performed using PECs from 3 mice per genotype per time point, and each experiment was repeated at least once using PECs from different mice, with similar results. Comparison between genotypes: p = NS.

PEC fractions from tumor-bearing WT and p47*^phox−/−^*mice were co-cultured with anti-CD3/B7.1-stimulated CFSE-loaded splenocytes from non-tumor-bearing WT mice (E∶T ratio 1∶1), and CD4^+^ and CD8^+^ T cell proliferation was assessed by CFSE dye dilution. CD11b-enriched PECs from day 42 and day 90 tumor-bearing WT and p47*^phox−/−^*mice completely suppressed anti-CD3/B7.1-stimulated CD4^+^ and CD8^+^ T cell proliferation (Figure 6). The suppressive effect of the CD11b-negative PEC fraction (principally composed of tumor cells) was variable; PECs from day 42 incompletely suppressed anti-CD3/B7.1-stimulated T cell proliferation while day 90 PECs had no effect (Figure 6). These results show that the myeloid (CD11b^+^)-enriched PEC population from tumor-bearing mice inhibit anti-CD3/B7.1-stimulated T cell proliferation independently of NADPH oxidase.

**Figure 7:**
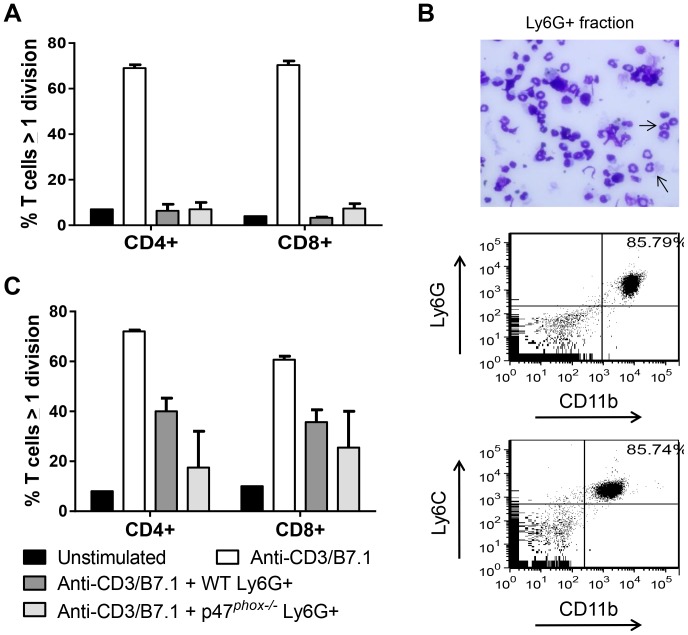
Peritoneal and splenic granulocytic MDSCs from tumor-bearing mice suppress T cell proliferation independently of NADPH oxidase. Ly6G-enriched PECs and splenocytes from MOSEC-bearing WT and p47*^phox−/−^* mice (day 90) were co-cultured with splenocytes from non-tumor-bearing WT mice (E∶T ratio: 1∶1). A) Ly6G-enriched PECs completely suppressed anti-CD3/B7.1-stimulated CD4^+^ and CD8^+^ T cell proliferation. PECs from 3 mice per genotype were evaluated. B) In Ly6G-enriched splenocytes, the majority of cells had a granulocytic morphology (arrows), and 86% of CD11b^+^ cells expressed granulocytic MDSC markers (Ly6G^+^Ly6C^low^). C) Ly6G-enriched splenocytes from WT and p47*^phox−/−^* mice modestly suppressed anti-CD3/B7.1-stimulated CD4^+^ and CD8^+^ T cell proliferation. N = 3 mice per genotype were used in this experiment, and results are representative of 3 experiments. Comparison between genotypes: p = NS.

We next evaluated the effect of NADPH oxidase on the suppressive function of the granulocytic MDSC (Ly6G^+^)-enriched PEC population. Because of the limited number of this population, functional studies were restricted to granulocytic PECs from day 90 after MOSEC administration. Ly6G-enriched PECs from WT and p47*^phox−/−^* mice completely abrogated anti-CD3/B7.1-stimulated proliferation of naïve CD4^+^ and CD8^+^T cells (Figure 7A). A limitation of these experiments is that Ly6G column purification led to enrichment of granulocytic cells, but not a pure population (Figure 5C).

To determine the role of NADPH oxidase in the function of systemic granulocytic MDSCs, we evaluated the suppressive effect of Ly6G-enriched splenocytes from MOSEC-bearing WT and p47*^phox−/−^* mice harvested at day 90. Following column purification, the majority of cells in the Ly6G-enriched fraction had a granulocytic morphology, and 86% of CD11b+ cells expressed granulocytic MDSC (Ly6G^+^Ly6C^low^) markers (Figure 7B). Co-culture experiments showed that the splenic Ly6G-enriched fraction from WT and p47*^phox−/−^* mice similarly suppressed anti-CD3/B7.1-stimulated proliferation of T cells (Figure 7C). Taken together, these results show that functionally suppressive granulocytic MDSCs accumulate in the local peritoneal microenvironment and systemically in tumor-bearing mice independently of NADPH oxidase.

## Discussion

Using genetically engineered NADPH oxidase-deficient mice, we found that NADPH oxidase did not affect tumor progression in murine EOC. The accumulation of MDSCs locally and systemically was similar in tumor-bearing WT and p47*^phox−/−^* mice. Although MDSCs from tumor-bearing WT mice had functional NADPH oxidase, the suppressive effect of MDSCs on *ex vivo* anti-CD3/B7.1-stimulated T cell proliferation was NADPH oxidase-independent. Together, our results show that MDSC accumulation and immunosuppression in murine EOC is NADPH oxidase-independent. We speculate that factors inherent to the tumor, tumor microenvironment, or both determine the specific requirement for NADPH oxidase in MDSC accumulation and function. Since modulation of redox status is a potential therapeutic approach to limit or overcome MDSC-mediated immunosuppression in cancer, it is important to understand NADPH oxidant-dependent and –independent pathways that promote MDSC development.

Oxidative stress by activated monocytes, neutrophils, and MDSCs is considered to be one of the components of the chronic inflammatory environment that suppress T cell function. Consistent with this notion, CD8^+^ cells transduced with catalase (which depletes hydrogen peroxide) had enhanced viability and CTL function [39]. Prior studies have shown that ROI generation is one of the main characteristics of MDSCs from tumor-bearing mice [4], [40]–[42]. Additional studies point to a direct role for the phagocyte NADPH oxidase in promoting MDSC accumulation and function [22], [23]. Corzo et al. [23] showed that splenic MDSCs from mice administered subcutaneous tumors had high levels of ROI generation and increased expression of NOX2 subunits, predominantly in gp91*^phox^* and p47*^phox^* compared with immature myeloid cells from naïve mice. In tumor-bearing mice, gp91*^phox^* was required for MDSC-mediated suppression of ex-vivo antigen-stimulated T cell proliferation and IFN-γ production [23]. These findings differ from our findings in murine EOC in which MDSC accumulation and function were NADPH oxidase-independent. Thus, rather than NADPH oxidase being uniformly required for MDSC accumulation and function, these findings point to factors inherent to the tumor and/or inflammatory microenvironment determining whether NADPH oxidase is required or dispensable.

There are several potential differences in experimental design that might account for NOX2-dependent and –independent MDSC accumulation and function. One possibility relates to the use of p47*^phox−/−^* mice in our studies versus gp91*^phox−/−^* mice used by others [22], [23]. Both p47*^phox^* and gp91*^phox^* are required components of NOX2 in both humans and in mice. Deficiency of either component leads to chronic granulomatous disease in humans [21] and to a consistent phenotype in engineered mice characterized by impaired host defense and excessive inflammatory responses to certain microbial products [28], [31], [35], [43], [44]. We found that p47*^phox−/−^* MDSCs, although completely deficient in stimulated NOX2 activity, had T cell suppressive properties similar to WT MDSCs. Still, we acknowledge the potential for these phox constituents to have NOX2-independent signaling that could affect MDSC accumulation and function. In tumor-bearing mice, MDSCs secrete and are induced by the myeloid-associated S100 proteins [7], [22], [45], which can prime NOX2 activity. Cheng et al. [22] reported that S100A9 expressed by hematopoietic progenitor cells inhibited their differentiation to DCs and macrophages and induced generation of MDSCs via a gp91*^phox^*-dependent pathway. The lack of effect of NOX2 in MDSC accumulation in our studies indicates that, at least in the MOSEC model, MDSC generation *in vivo* is NOX2-independent. Other factors that could influence the requirement for NOX2 in MDSC differentiation relate to differences in tumor-derived products (e.g., secreted growth factors, cytokines, chemokines, and angiogenic products) and the local tumor microenvironment (e.g. peritoneal fluid versus subcutaneous). We believe that a strength of our approach is that we focused on MDSCs in the ascites of MOSEC-bearing mice – which are likely to be the most relevant in modulating anti-tumor immunity in the local tumor microenvironment.

Both tumor-derived factors and products produced by non-tumor cells in the microenvironment, including G-CSF, GM-CSF, cytokines (e.g., IL-1β, IL-4, IL-6, interferon-γ), S100A8/A9, cyclooxygenase-2 and prostaglandin E2, indoleamine 2,3-dioxygenase, and ROIs can promote MDSC development and/or immunosuppressive activity [22], [24], [45]–[49]. Because MDSCs can be induced by multiple factors, it is possible that no single molecule is essential for generating MDSCs [1]. In addition, the lack of effect of NOX2 on MDSC accumulation and function does not rule out an effect of other sources of ROIs. There are NOX2-independent sources of ROI generation, including xanthine oxidase [50] and mitochondrial ROIs [51] that have antimicrobial host defense capacity and, conceivably, could contribute to MDSC generation and/or function in the absence of NOX2. These NOX2-independent pathways could potentially react with nitrogen intermediates to generate peroxynitrite, which is required for nitration of TCR/CD8 and induction of T cell tolerance [4]. As a precedent for this concept, the interaction of xanthine oxidase and reactive nitrogen intermediates overcame the requirement for NOX2 as a mediator of ROI-mediated acute lung injury [52]. We speculate that NOX2 and other ROI-generating enzymes represent alternative pathways for ROI generation that can prime the development of MDSCs. Further work using small molecule inhibitors and genetically engineered mice with deficiencies in specific ROI-generating pathways will be required to test this concept.
